# Family Digital Literacy Practices and Children’s Mobile Phone Use

**DOI:** 10.3389/fpsyg.2016.01957

**Published:** 2016-12-23

**Authors:** Melody M. Terras, Judith Ramsay

**Affiliations:** ^1^School of Media Culture and Society, University of the West of ScotlandPaisley, UK; ^2^Department of Interdisciplinary Studies, Manchester Metropolitan UniversityCrewe, UK

**Keywords:** smartphones, psychology, parenting styles, home environment, children, parental use of technology, internet

## Abstract

Smart phones are ubiquitous in everyday life and are having a major impact on work, education, social relationships and modes of communication. Children are the fastest growing population of smart phone users, with use often focusing around internet access, e.g., 1 in 3 internet users in the UK are under 18 years of age. Despite their widespread use, relatively little is known about the factors that underpin children’s use. The home is a significant ecological context of development and recent research has highlighted the importance of the home environment in promoting and supporting the development of both safe and unsafe online behavior. Yet the importance of these influences currently remains relatively unrecognized. Therefore, in this paper we present a narrative review of evidence examining parental practices concerning digital communication technologies and applications, with a particular focus on smartphones, and how they relate to the use of technology by their children. Emerging evidence to date indicates that two important factors are at play. Firstly, parental technology use is closely related to that of their child. Secondly, that despite parents frequently voiced concerns about the nature and extent of their child’s mobile phone use, parents themselves often engage in a number of unsafe internet behaviors and excessive phone use in the home environment. Our review identifies two crucial lines of enquiry that have yet to be comprehensively pursued by researchers in the field: firstly, the adoption of a psychological perspective on children’s emergent behaviors with mobile devices and secondly, the influential role of context. Given parental concerns about the possible negative impact of technologies, parental awareness should be raised about the influence of their behavior in the context of internet safety along with the adoption of good digital literacy practices. It is anticipated that a comprehensive characterization of the associated contextual factors influencing smartphone use will serve as a catalyst for debate, discussion, and future research.

## Introduction

Smartphones are ubiquitous in everyday life and influence work, education, family and social relationships, and modes of communication. Smartphone ownership is growing apace and smartphones are rapidly becoming the dominant mode of internet access: in the UK there has been almost a threefold increase (6–16%) in a year in the use of a smartphone to access online resources (Ofcom, 2016a). Children are the fastest growing population of smart phone users, with use often focused on internet access, e.g., 1 in 3 internet users in the UK are under 18 years and 11% of 3–4 year olds are already internet users (Ofcom, 2013). The rapid pace of the technological development and use of smartphones offers major challenges to researchers aiming to understand the personal and societal impact; as well as policy makers aiming to maximize the benefits and minimize the risks (Livingstone, 2016). If these concerns are to be adequately addressed, a detailed understanding of the interwoven contextual, demographic and psychological factors that influence smartphone use is required.

Until recently the majority of research had focused on mobile and smartphone use by adults, and neglected the experiences of the increasing numbers of children who own and use a smart phone. More recently, however, a study examining the socio-demographics factors in the adoption and use of smartphones in children aged 9–16 years across a number of European countries has demonstrated the complex interaction of socio-demographic factors that are strongly influenced by context, at a number of levels: parent/family, societal and cultural/country level: these impact not only ownership but more importantly use (Livingstone et al., 2015a). Of particular relevance is the related influence of parental use of a smartphone and the extent of parental monitoring and control concerning their child’s smartphone use, e.g., the type of data plan provided, the extent of domestication of the internet, age of first internet access and the range of opportunities available for mobile internet access (Mascheroni and Ólafsson, 2015). These findings remind us of the complexity of influences that help shape mobile phone behavior and it is a detailed consideration of these factors that is the focus of this paper. Specifically, we explore the factors that influence children’s use of smartphones in the ecological context of the home. For children the home environment, especially parental behavior, may exert a significant influence. Therefore, this paper provides a comprehensive review and synthesis of the literature on parental attitudes and practices, especially family digital literacy practices, and considers how they influence the use of smartphones by children. In particular, we examine the strengths and weaknesses of the existing evidence base and offer suggestions, at the level of both theory and practice, for how the field may move forward.

## The Home: A Significant Context of Development

Although, the ease of internet access offered by smartphones affords children the potential to experience rich digital lives by providing new contexts of development and being; the home, especially for young children, remains a significant influence on children’s cognitive and psycho-social development in general and their use of technology in particular (Plowman et al., 2012). Recent research highlights the importance of considering parental use and family practices concerning the use of technology such as computers, tablets, mobile phones, and games consoles within the home (Stephen et al., 2013; Marsh et al., 2015). Of particular importance is the emerging evidence indicating that parental technology use, especially the use of smartphones to access social media is closely related to, and in many instances predictive of, that of their child (Terras et al., 2016). Therefore, it is essential that the developing evidence base on smartphone use recognizes the significance of these influences. In order to build a comprehensive understanding, the evidence base needs to be multi-disciplinary in nature as it is only by recognizing and synthesizing theory and research from a number of domains that a full understanding will be achieved. Given that the central aim is to understand and predict behavior, then psychological input should make a major contribution.

Developmental psychological theory and research has long recognized the importance of context, especially the home, and the behavior of those within it, in supporting cognitive and social development (Vygotsky, 1978). Bronfenbrenner (1979) argued that development occurs within a number of embedded ecosystems that influence the child either directly or indirectly. The home environment is one such ecosystem which has a direct influence on a child. Such contextualized theories indicate the importance of considering the psychological character of the individuals, social class, parenting practices and values to ensure ecological validity and capture the socio-cultural practice of the home (Tudge et al., 2006; Hedegaard, 2009). In this digital age, these practices reflect attitudes, preferences and the use of wireless devices to access the internet and Web 2.0 applications for social and educational uses. Therefore, we begin our review with evidence of how parenting styles, parental preferences and use, and the technological ethos of the home influence a child’s smartphone use.

### Parenting and Internet Mediation

A significant body of psychological evidence informs our understanding of the influence of parental practice on child development. For example, Bowlby (1969) advanced our understanding of the nature and function of the bond between parent and child, and how the security of this bond determined the child’s consequent “attachment style” which not only characterizes the parent–child relationship but also generalizes to other relationships, life satisfaction and well-being across the lifespan (O’Connor and Scott, 2007; Ciairano et al., 2008). Of major significance is the work of Baumrind (1966) who identified four major parenting styles: authoritative, authoritarian, permissive and laissez-faire depending on how they fall on the two orthogonal dimensions of warmth/involvement and control/demands. Permissive and authoritative parents are both high on parental warmth and control, with permissive parents placing relatively low demands. Parents who adopt an authoritarian or a laissez-faire style tend to be low in terms of warmth and involvement, but vary in terms of control with authoritarian parents exerting high levels of demand and control. Authoritative parenting is associated with superior academic achievement and a positive social orientation, whereas children of authoritarian parents tend to have the poorest adjustment (O’Connor and Scott, 2007).

Recently, Baumrind’s classification of parenting styles has been extended to capture parental practices concerning internet use (Valcke et al., 2010). This extension reflects how the home environment is evolving in this technological age, and how parents are adapting their parenting practices to suit. Addressing the challenges brought by technology is not new, with television being a prime example. However, the increasing proliferation and enthusiastic uptake of Web 2.0 applications, on-demand entertainment and internet access facilitated by wireless devices such as tablets and smartphones is exerting a strong influence within the home (Terras et al., 2016). Smartphones offer specific challenges to parents as the decreasing size of smart handheld devices means their use is less obvious and easier to conceal, and the ability to regulate internet access by technological filters is determined by the user’s digital literacy knowledge and skills (Vincent, 2015).

Parents attempt to manage the challenges presented by smartphones, especially the internet access they support, through the process of parental mediation which can be defined as “the diverse practices through which parents try to manage and regulate their children’s experiences with the media” (Livingstone et al., 2015b, p. 7). Evidence indicates that the four parenting styles identified by Baumrind influence both the nature and number of internet mediation strategies used. Livingstone et al. (2011) identified five main types of parental mediation to regulate internet and mobile media across 25 European countries of children aged 9–16 years: (1) Active mediation of internet use: characterized by discussion of internet content and online activities and conjoint online activity; (2) Active mediation of internet safety to promote safe internet use; (3) Restrictive mediation which involves the limiting of online access in terms of time, activities, content and use; (4) Technical restrictions characterized by the use of technological filters to restrict and monitor online activities, and (5) Monitoring which reflects *post hoc* checking of internet activities. Active mediation of use and safety are the most frequently used strategies for children aged 9–16 years, with more restrictive practices being used for younger children. Regardless of age, girls tend to receive higher levels of parental mediation than boys (Livingstone et al., 2011). Active and restrictive mediation tend to be the most effective ways of maximizing online opportunities and minimizing the experience of online risks (Dürager and Livingstone, 2012; Mascheroni et al., 2013).

The use of a discursive mediation strategy, a key characteristic of active parental mediation, is also a common strategy used in the US (Clark, 2013). Supportive communication between parents and early adolescents has been shown to offset the negative effects (e.g., reduced life satisfaction) of using electronic media (Boniel-Nissim et al., 2015). Moreover, van den Eijnden et al. (2010) report that “qualitatively good communication” (p. 77) about internet use on the part of parents decreased the likelihood of their adolescent children developing compulsive internet-related behaviors. Monitoring and negotiating access to digital media has had a degree of success: in a study of children reaching the age when they would go to secondary school, most had developed their own “repertoire” (p. 333) of use and understood that this particular form of autonomy emerged from accommodating parental rules (Davies, 2011). Smartphone applications such as *FamiLync* have recently been developed to support families in jointly regulating the time they spend online by creating a common mechanism for understanding usage behaviors: use of this application meant that children were more likely to actively participate and the use of smartphones decreased (Ko et al., 2015).

As noted earlier, culture is an important contextual factor that shapes behavior and this influence is clearly reflected in parenting practices: the extent of parental mediation varies widely across European countries, with parents in Ireland, UK, and most central and European countries reporting the use of restrictive mediation strategies (Helsper et al., 2013). Parents from Northern European countries tend to use active mediation; whilst parents from Eastern European countries fall into two groups (i) those who mediate less than other parents or (ii) parents who do mediate tend to use a range of strategies (Helsper et al., 2013).

To summarize, at a practical level existing evidence indicates that there is a wide range of strategies, both formal and ad hoc, that are being adopted by parents to manage their children’s access to, and use of digital devices. At a theoretical level, the preceding discussion illustrates the insights that can be gained through the application and extension of existing theoretical explanations of parental practices into the area of smartphone behavior. However, further research, particularly longitudinal studies is required to ascertain the long term effectiveness of parental practices and understand the skills and practices that support efficient management within the home. Cultural differences in parental mediation practices are also evident and the emergence of these issues is a timely reminder that despite the global nature of technological development, variation and differences at the level of the individual and the wider social and cultural contexts in which they operate remain and require further investigation.

## Family Digital Literacy Practices

The previous discussion illustrates how parents apply their preferred approach to parenting to help regulate the extent and nature of their child’s use of technology. Not only is technology changing, but the skills and attitudes of parents themselves are evolving as they gain technological experience and as the so called *digital natives* become parents themselves. How is this influencing parenting and the use of technology within the home? It is essential to consider not only whether parents adopt a protective or an enabling approach to their children’s digital use, but also why they do so – what attitudes, preferences and concerns determine these choices? Insight may be derived from the emerging literature on Family Digital Literacy Practices which can be defined as the way(s) in which children and their families interact to jointly shape their behaviors with digital media (Sefton-Green et al., 2016).

### The Home Technology Environment

Children, even very young ones, are experiencing a technologically rich home environment. Research in the UK involving 346 parents of children aged 3–5 years indicates that children experience a range of technologies at home such as toys that act as mobile phones and computers, games consoles, and engage in a range of techno-cultural activities such as speaking on the telephone and taking pictures of pets (Plowman et al., 2010, 2012). This enables children to learn how to use technology, as well as developing their knowledge of the world from the content of the media accessed (Plowman et al., 2010, 2012).

The touchscreen interface of tablets make them popular devices for pre-school children (age 0–5 years), with one quarter of children under three and one third of 3–5 year olds owning their own tablets and frequently using them to watch videos, play games, and draw (Marsh et al., 2015). Parental control is easier to exert with younger children and parents tended to use tablet applications to reinforce good behavior. Children are more likely to use a tablet together with a parent, than alone; with the decision to use the tablet generally residing with the parent (Marsh et al., 2015). Smartphones also draw upon touch screen technology and further research is required to establish whether the current use of tablets will generalize to smartphones. Much of this generalization will depend upon the nature of the home environment.

It is clear that children are exposed to, and engaged with, technology from a very young age and parents are playing a significant role in not only providing, but also enabling its use. Stephen et al. (2013) identified four dimensions within the home environment that distinguish children’s experiences with technology: (1) family attitude toward technology as an educational support, (2) parents’ attitudes toward enabling children’s education, (3) the presence of siblings, and (4) demands on parental time and individual differences. These dimensions reflect the mediation styles and attitudes and preferences of parents and children and clearly demonstrate how parental practices help define the socio-technological ethos of the home, which in turn influences the use and preferences of the child. Recent evidence indicates that the home technology environment cannot only be shaped by parents, but that children themselves can play an active role in influencing the behaviors of family members by acting as “brokers” in the adoption of digital media in the home environment (Correa, 2014, p. 111). Further research with larger more representative samples is required to clarify the nature and processes that determine not only parental but also child influences within the home.

### Parental Influences and Child Technology Use

Parents own use of media, rather than their attitudes concerning their child’s use, may be a key determinant of children’s use: a study of 2,300 parents of children aged 0–8 years, indicates that parental time spent using television, computers, smartphones, and tablets was associated with their child’s “screen time” (Lauricella et al., 2015). Although, setting limits on “screen time” is a common internet mediation technique, it may be detrimental: a survey of 735 mothers suggests that always setting time limits on “screen time” may increase the amount of time spent using smartphones, computers and games consoles (Kesten et al., 2015). This may be attributable to children maximizing their usage time. Therefore, parents need to fully appreciate the potential consequences of their mediation practices as well as their own behavior.

Parental attachment style also influences children’s smartphone behavior. A study of 1808 high school students in Taiwan indicated that weaker parental attachment was associated with internet addiction and cyberbullying (Chang et al., 2015). Evidence also indicates a relationship between parental depression and internet addiction in adolescents in a study of 1098 parent-and-child dyads in Hong Kong (Lam, 2015). In a longitudinal study of Korean children, “higher democratic parenting” (p. 1) (defined as being warm, with supervision and rational explanations) was associated with less addictive use of smartphones (Bae, 2015).

Homework activities is one area for collaboration between parents and children based around technology use. Whilst formal schooling may provide a foundation for digital literacy skills in children, the influence of parents’ own use of technology within the home complements their children’s formal learning. However, a major barrier to the development of shared practices is parents’ potential lack of basic digital skills. It is no longer sufficient to be able to read a book and write with a pen: one also needs to be able to access and read information online and respond appropriately. These abilities are captured by the concept of Digital Literacy that can be defined as “The skills, knowledge and understanding that enables critical, creative, discerning and safe practices when engaging with digital technologies in all areas of life” (Futurelab, p. 8). Despite a number of global policy initiatives such as The United Nations Educational, Scientific and Cultural Organisation’s (UNESCO) work on media literacy and similar work by the telecommunications regulator in the UK (Ofcom), 23% of UK adults do not possess basic digital skills (Go UK, 2014). Given the influence that parents exert over children, this skills gap requires urgent attention if children are to learn how to use new technologies responsibly and safely in their home environment.

There is evidence to suggest that parental digital technology use is not only associated with, but also predictive of their child’s use (Terras et al., 2016). The existence of an identifiable relationship between parent and child “screen time” is important as it has been demonstrated that the amount of time spent using screen-based digital devices has implications of well-being. Lower depression levels are associated with lower “screen time” (for leisure purposes) in both children and young adults (Kremer et al., 2014), and higher levels of social media use are associated with lower happiness levels (Brooks, 2015), and increased depression and sleep disturbance in young adults (Levenson et al., 2016; Lin et al., 2016).

In summary, there is clear evidence that the use of mobile digital devices is deeply embedded within the many informal familial contexts that a child inhabits, and that their use of these devices is influenced by the practices of the other individuals who co-inhabit those spaces. These include parents, siblings, classmates and friends and their influence may be exerted in a number of ways, some of which may be deliberate, however, some are not. Parents often underestimate the powerful influence their use of technology has on their child (Plowman et al., 2012). The question then is why does this happen? A frequent form of learning used by children is observational learning where behaviors are observed and then imitated (Bandura, 1977). This form of social learning has clear practical implications for the use of technology in the presence of children, as it indicates that they may potentially model their own technology-related behaviors upon those around them.

### Smartphone Use, Attitudes, and Concerns

To fully understand the home technology environment and associated family practices, it is necessary to examine how and what range of technology children use within the home. At the time of writing, 1 in 3 internet users are under 18 years of age (Livingstone et al., 2015a) and in the United Kingdom (UK), 11% of 3–4 years are internet users (Ofcom, 2013), 28% of 3–4 year olds use a tablet at home (Formby, 2014), with children aged 2–4 demonstrating “multimedia, multimodal practices” (Marsh et al., 2015). A survey of European Union children age 9–16 years indicates that these practices include watching video clips (76%), playing games (83%), instant messaging (62%), social networking (82%) and using the internet for homework (85%) (Davies, 2011).

Although such findings offer a snapshot of current practices, it is necessary to move beyond a simple descriptive level to a more explanatory one. To obtain a complete understanding of the influences that shape children’s use and family literacy practices it is essential to understand the psychological drivers for smartphone use. It is necessary not only to quantify the scope and scale of technology used, but also to understand the function that technologies serve and their perceived role in daily life. These issues are only starting to be addressed but the emerging research is offering some interesting insight. For example, Vincent (2015) identified six themes which reflect children’s use of smartphones. Children perceive smartphones as offering a number of ways of *Being*: *Mobile, Social, Educated, Entertained, Me* (self-identity) and Protection/privacy. The conceptualization of smartphones enabling children to *Be Mobile*, is interesting as children’s experience of being mobile is not simply the idea of anytime anywhere access, it is more a case of using this aspect of connectivity to allow them to participate in both online and oﬄine activities at the same time. The second theme of *Being Social* captures the range of communication and social networking opportunities supported by smartphone internet access, with children expressing the view that smartphone ownership has helped them to become more sociable with smartphones being viewed as essential by many children to help support their social life. Thirdly, the educational potential of smartphones was also recognized as the mobile internet offers on demand access to a range of formal and informal informational resources and supports the development of media literacy skills. Fourthly, smartphones also offer a range of resources that allow children to *Be Entertained*, interestingly the risky nature of the potential content that can be accessed is recognized by children. Fifthly, smartphone use, especially the opportunities it allows for communication and identity development helps promote the development of self-identity and social well-being- it allows children to *Be Me*. Many children reported a strong personal and emotional connection to their smartphone as this device allows them to engage in a range of activities that support their self-expression and identity. Finally, smartphones, despite the potential risks associated with online activity, were viewed as a means to ensure safety as it allows contact with parents if necessary.

Although children are generally positive about smartphones, as they are about the internet in general, they also recognize the strengths and limitations of different types of technology. A major disadvantage of a smartphone is screen size and the absence of a keyboard, so they tend to select the best device for the task (Haddon and Vincent, 2015). Children were of the opinion that smartphones had increased peer communication and recognized how the pressure to respond quickly influences the nature of the response. Children, like their parents, were more concerned about the risks associated with the internet rather than the smartphone itself. Although the potential for smartphones to increase the risk of cyber bullying was acknowledged by children, most concern was expressed over identity theft as this can happen relatively easily if someone takes/uses your smartphone pretending to be you (Haddon and Vincent, 2015).

It is also essential to understand parents’ perceptions of smartphones as this will aid understanding of why certain parental mediation strategies are being used and promote the development and use of the most appropriate and effective strategies to meet their needs. A major concern for parents is the financial cost of smartphones and this leads parents to impose restrictions on use and limits on Apps provided, especially those which include within-app purchases. Interestingly, children too are conscious of usage costs and often set limits on their own use (Haddon and Vincent, 2015). Parents also expressed concern that smartphones would increase “screen time” more generally, thereby limiting the range of other experiences, especially social experiences, which parents are keen to promote. Concern about the potential detrimental impact of online socializing on oﬄine social behavior was a major motivation for limiting “screen time.” Parents tend to be more concerned with online risks generally and do not view smartphones as a specific risk in their own right. It is the internet access and time spent online that smartphones offer, rather than the device itself, that causes parents’ most concern (Haddon and Vincent, 2015).

In summary, the relationship between parent and child is deeply situated and subject to myriad influences. Indeed, from a psychological perspective, it can be characterized as essentially a socio-cognitive relationship. Such a relationship is comprised of social elements and psychological elements – each different and each playing an influential role. Framing parent–child technology-related relationships and behaviors in this way means that we can consequently draw upon well-established psychological knowledge and mechanisms to describe and explain children’s emergent behaviors in new digital home contexts. To date the majority of the existing research concerning children’s smart use tends to be a-theoretical in nature; hence the adoption of a more defined theoretical framework that could help identify and define future research would be beneficial to progress the field. The need for such a framework becomes all the more apparent when we consider the rapidly evolving nature of the home technology environment.

### The Evolving Home Environment in the Digital Age

The home context not only shapes children’s use of technology, the use of technology within the home is also influencing the nature and social dynamics of the home contexts itself. Parents have reported using technology to negotiate with and to manage children. For example, Wartella et al. (2013) found that parents used technology as a new bribe to manage children when they were busy (this arguably suggests a disengaged style of parenting), with the potential consequence that, at worst, children might potentially be at risk of behavioral problems or socioemotional problems (O’Connor and Scott, 2007). Interestingly, parents who do not consider technology as making parenting easier, viewed technology negatively and expressed the opinion that it had a detrimental influence on children’s social skills (Wartella et al., 2013). Zhu et al. (2015) found that the quality of relationship between parent and child was associated with addiction to internet games through poorer school connectedness and “affiliations with deviant peers” (p. 159). Individuals who demonstrated problematic mobile phone use (as measured by Bianchi and Phillips’ 2005 scale) cope less well with the distractions generated by digital technology. Recent evidence in adults suggests that cognitive resources, particularly low working memory capacity and poor attentional control, are factors worth exploring in future research concerning potential problematic mobile phone use by children (Hadlington, 2015).

Consistent with a contextualized approach, children may also influence their parent’s media use, with research indicating that the transfer of technological knowledge concerning computers, mobile phones and the internet may not only operate in a top–down fashion (from parent to child) but also in a bottom–up manner: from child to parent (Correa, 2014). Findings indicate that the active sharing of knowledge is influenced strongly by traditional demographic factors such as gender and social class, i.e., women/mothers are more likely to gain knowledge from their children and the influence of child knowledge is greater in families with lower socioeconomic status. Interestingly parenting style also influences the process, with child knowledge less likely to be shared in families where an authoritarian style of parenting is used (Correa, 2014).

Smartphones are also influencing the nature of parent–child communication. A study of 1322 parents in the United States who used text messaging, email, social media and videoconferencing (Skype), illustrates that the older the child, the more technology-mediated communication was used. Smartphones also support communication between parents, with text messaging being extensively used between co-parents when they had a school-aged child (Rudi et al., 2015).

There is a corpus of research indicating that technology may adversely influence the nature and quality of parent–child relationships (Nie et al., 2002; Subrahmanyam and Greenfield, 2008; Chen et al., 2010). In particular, it has been argued that technology could potentially assume the role of an attachment figure for children (Lei and Wu, 2007; Chen et al., 2010) thereby acting as a barrier to children forming attachments in the traditional manner. Research also indicates an association between poor quality maternal childhood attachment and mobile phone dependence (Toda et al., 2008). More recently, research in young adults has examined how attachment styles within human relationships may generalize to the relationships individuals develop with their smartphones: with anxiously attached individuals more likely to display anxious attachment in relation to their smartphone use (Konok et al., 2016).

With regards to managing these potentially negative influences, there is a persuasive case for parents putting their phones and tablets out of sight as the mere presence of a smartphone influences social interactions. Having a mobile phone within one’s line of sight can influence the perceived relationship quality and this detrimental effect is strongest when discussing topics of greater rather than less importance, with reduced empathy being displayed and less trust being experienced (Przybylski and Weinstein, 2013). Therefore, to increase the quality of the parent–child relationship, it is advisable to remove devices from sight when not in active use. The quality of family connections can also be increased by more frequent family mobile phone use, joint television watching and joint video game playing (Padilla-Walker et al., 2012).

In summary, not only do the practices of family members influence children’s device use, children’s use and knowledge of technology may also that of their parents. The evidence also indicates that mobile digital technologies themselves are to an extent, if not supplanting, then at least complementing the relationships that children have with their parents, siblings, and others. It is clear that technologies such as smartphones have become embedded within the home environment and have become instrumental in not only supporting parent and child relationships, but that their use is also influenced the nature of that environment and the relationships within it. Hence, further research is required to help determine the processes through which this occurs.

## Understanding Contextual Influences

From our review of the literature two major themes are evident, Firstly, the home environment, especially parental attitudes and use of technology generally, exerts a strong influence on that of their child. Secondly, that despite parents frequently voiced concerns about the nature and extent of their child’s mobile phone use, parents themselves often engage in a number of unsafe internet behaviors and excessive phone use in the home environment and seem relatively unaware of the potential influence of their behavior in implicitly setting standards and validating practices. The issue then is how well does existing evidence based explain these findings and what questions remain unanswered and require further research?

### Assessing the Current Evidence Base

From our review of the literature, it is clear that there are a number of distinct forces at work that shape children’s emergent behaviors with new technologies in the home. In general they can classified into two main areas: context and individual differences. It is essential to remember that these influences are not distinct, and in practice they frequently interact to influence smartphone behavior.

Children engage in a myriad of context(s) that may help shape their use of technology. Within these contexts there are physical and material factors such as the nature, range and availability of technology and opportunities for internet access. Contexts cannot only be characterized in terms of their material resources but also in terms of the social and informal learning opportunities they provide, such as enabling attitudes to technology use. Within the home and indeed beyond the home environment, children interact with many individuals, whether they be caregivers, relatives, siblings, neighbors, friends, classmates, or others. It is important to recognize that all these individuals vary in terms of their attitudes, preferences and skills concerning technology all of which can contribute to a child’s use and understanding of technology. It is also essential to recognize that like technology itself, each individual’s attitude can vary across time and across contexts, generally as the result of increased experience and exposure to a wider range of opportunities. Specifically, physical changes such as moving house, along with changes to the nature of the family, e.g., the arrival of new siblings influence media habits (Ofcom, 2016b), along with developments that are internal to the child, e.g., age-based cognitive development (Ofcom, 2016b).

Yet despite their importance, research to date has only focused on a limited number of contextual factors and individual differences, with research generally reflecting traditional demographic factors such as age, gender and social class. A notable exception is the consideration of differences in terms of parenting style and its associated implications for internet mediation. Much of this research is based on existing psychological theory and research and greater use of psychological insights may promote increased consideration of the wider range of individual differences in personality, motivation, and the attitudes and cognitive abilities that underpin the skilled use of technology.

Much of the existing evidence based is limited by methodological weakness such as small and unrepresentative samples. These are major limitations given the high level of variability across individuals and contexts. It is also essential that future research captures changes across time. It is clear that parenting styles evolve and consequently, parental approaches to managing technology use by their children may also develop over time. The lack of longitudinal studies and small sample sizes makes it difficult to determine the nature of changes and the relative extent of the influence of different contextual influences across time. It also hinders the identification of the mechanisms and processes through which this influence is exerted, i.e., whether they function as mediators or moderators (e.g., Hui-Lien et al., 2016) on a child’s smartphone use. A full understanding of these influences is essential in order to maximize the benefits and minimize the risks of smartphone use.

A further methodological weakness concerning sampling is the relatively wide age range of children sampled. Children are not a homogeneous group and the tendency to treat them as such is masking important age related differences. For example, recent research from the Ofcom Children’s Media Lives Longitudinal study in the UK highlights the benefits of longitudinal research and more detailed analysis of technology use by age group. For example, adopting a wide age range of 6–15 years indicates that smart use accounts for 20% of their use of all technological devices per week. However, when a finer grain analysis is used the allocation of time is different across age groups, with smartphone use only accounting for 6% in the 6–11 year category but 35% in the 11–15 years age group; much higher than the reported adult (16 years +) weekly use of 20% (Ofcom, 2016b).

### The Importance of Adopting a Psychological Perspective

Many of the deficiencies in the existing evidence base, especially those concerning the lack of detailed consideration of a wider range of individual differences and contextual factors may be attributable to a lack of detailed and explicit consideration of psychological factors within the general literature on children’s smartphone use.

Research effort over the past century in the field of Psychology indicates that human behavior is influenced by contextual factors: behavior does not occur in a vacuum, rather, it is shaped by, and in many instances also shapes, the environment in which it occurs. This theme is apparent across all sub-branches, be it behaviorist approaches which emphasizes the importance of reinforcement and observation in shaping behavior, to cognitive and socio-cognitive approaches with their focus on specifying the internal constructs and processes that help us process information and make sense of the world around us. From a psychological perspective, it is inconceivable to consider de-contextualized behavior, so it is only prudent that the context, motives, preferences and reward of smartphone behavior are recognized. Consideration of contextual influences and the psychological factors that help to create and define them are paramount.

On the basis of our review and synthesis of the literature we have identified a range of contextual influences on children’s smartphone use such as parenting style and attachment, internet parenting style, awareness and the influence of parental behavior on children’s smartphone use. In doing so, we have gained insight into the motivations for smartphone use and developed a more nuanced understanding of the role that smartphones play within children’s daily life, i.e., they serve a number of socio-emotional functions that support the development and expression of self-identity and being. From a psychological perspective, smartphones enable children *to be* and develop across a range of educational and social contexts. As well of these benefits, children also recognize the negative aspects, both of the technology itself, and more importantly of the potential risks that internet access may bring. Interestingly, both children and parents distinguished between the internet itself and the device that permits access, with concerns about risk generally concerning the internet and its associated content, rather the smartphone itself.

Parents engage in a range of behaviors in an attempt to regulate and manage what they perceive as risks- excessive “screen time” and inappropriate content being of major concern. Existing research offers important insight into parental motivations and behavior, with evidence indicating that parental internet parenting style is a product of the interaction between psychological and demographic factors such as age, income and education. For example, parental mediation strategy is influenced by parental education: less warmth and control is often manifested by less educated parents, and less educated parents with low levels of digital skill tend to use technical restrictions and be less active and inconsistent in their mediation (Livingstone et al., 2015b). Consideration of psychological influences extends our understanding of the challenges parents experience in managing their child’s interactions with technology: developing a deeper understanding of children’s motives for use, will help inform parenting behavior and raise their awareness of the complex factors that influence both their behavior and that of their child. For example, excessive screen time emerged as a major concern for parents as they were concerned about the potentially negative effect it may have on a face-to-face social skills. Yet children do not share these concerns and report a range of social gains and become annoyed when technology is used in offensive ways in social situations. It seems that although parents are using smartphones and digital communication technology generally, their motives and the associated benefits in some instances may be different from those of their child. The use of joint technology-based activities and discussion as a key element of active internet mediation should be promoted as a means to inform parents about their child’s motives for smartphone use thereby ensuring that the management strategies they use.

### Future Research Directions

Viewing children’ smartphone use from a psychologically informed contextualized perspective not only deepens our understanding of the existing evidence base concerning parental and contextual influences within the home, it also highlights areas for future research. Further research examining parenting practices, especially the role played by fathers is required, as the vast majority of research to date has focused on mothers (Hessel et al., 2016). Additionally, a more fine-grained analysis of parental practices is required, that differentiates between parents use of technology generally and the use of technology for parenting (Walker and Rudi, 2014), with future research considering how these different uses of technology may influence parenting styles, the nature and degree of internet mediation and the digital ethos of the home in general. Understanding attitudes and preferences concerning online parenting is essential to inform the development of online parent education programs and interventions. The important role of siblings’ behaviors and attitudes toward digital device use requires further examination, to help develop a more comprehensive understanding of the role played by siblings within the home technology environment.

As noted earlier, demographic and psychological factors interact in a complex way to determine smartphone use. Although, smartphones have the potential to address inequalities in terms of access, individual differences in use and skills still remain (Donner et al., 2011). Although, Smartphones may help to narrow the divide in access by doing so they may also widen the usage gap. Emerging evidence from children and adults indicates that the increased use of smartphones as the sole means of internet access is detrimental to the digital literacy skills of those who only have smartphone access (Mascheroni and Ólafsson, 2015; Ofcom, 2016a). It is therefore essential that efforts to promote and support internet access via a range of a digital devices are maintained and the promotion on digital literacy skills in both policy and practice is retained and developed.

As noted earlier, the existing evidence base concerning children’s smartphone use is limited by methodological issues, lacks integration and is largely a-theoretical in nature. Although this is not entirely unexpected given the relatively new nature of the field, future research should address these omissions. For example, our review indicates that much of the concern about smartphones use is related to the internet access it facilitates. Hence insights can be gained from the evidence base concerning internet use, with future research benefiting from the integration of currently distinct domains of research such as the exploration of factors influencing uptake and use of technology typified by the use of the application of the Technology Acceptance Model (Davis, 1986), determinants of internet addiction (Burnay et al., 2015) and the use of both qualitative and quantitative methods with larger more representative samples to examine internet parenting style (Özgür, 2016).

Children’s growing use of smartphones to access online content, necessitates the consideration of these factors at an increasing younger age. Multidisciplinary working, drawing upon the expertise of psychologists, sociologists, technologists, product developers and policy developers amongst others, along with increased integration and convergence is required to develop a more nuanced understanding of the factors that influence smartphone behavior and the contexts in which it occurs. An interesting example of such convergence concerning the importance of context is exemplified by the sociological perspective of Correa (2014) which exams the influence of demographic family context on smartphone use and draws upon domestication theory to frame the roles of culture, home politics, and family members in the use of technology. As noted earlier, Culture is recognized as an important contextual influence as different parenting approaches and internet mediation strategies are used in different countries- here again a unifying framework is needed as existing research is fragmented with research generally being reported by the country, e.g., UK, Taiwan, Chile, with minimal attention being paid as to why these differences may occur.

However, future research needs to explore not only what we do with smartphones and the associated demographic influences, but why we do it, i.e., the nature of the behavior and the psychological function it serves. Such insight is beginning to emerge in relation to the problematic use of mobile phones, with recent research indicating that problematic use is influenced by a range of psychological factors such as high self-monitoring, extraversion and low self-esteem (Bianchi and Phillips, 2005; Kim and Hahn, 2015). Although this data is from adults, it is important to consider as it offers interesting insight into factors that may influence the development of problematic smartphone behavior by children. This may best be achieved by adopting a longitudinal perspective with large representative samples which will offer insight into the development of problematic behavior over time and support the application of robust statistical measures to help identify the moderating or mediating nature of these influences.

## Conclusion

In this paper we have reviewed the influences on children’s smartphone use in order to extend our understanding of the challenges parents experience in managing their child’s interactions with technology. Developing a deeper understanding of children’s motives for use, will help inform parenting behavior and raise their awareness of the complex factors that influence both their behavior and that of their child. A full understanding of the psychological factors that shape parental mediation of the digital media used by their children will inform policy and practice by helping to minimize risks whilst enabling children to gain maximal benefit from technology and the varied and exciting range of contexts and content the internet provides.

Our review indicates that the existing evidence base concerning children’s use of smartphones is currently limited by methodological weakness and a lack of integration across disciplines at the level of both theory and practice. Our review identifies two major omissions: (1) lack of explicit recognition of the insights that can be gained by viewing children’s smartphone behavior through a psychological lens; and (2) lack of explicit recognition of the complexity of the family technological context and the important influence it has on children’s use of smartphones.

Emerging evidence indicates that two important factors are at play. Firstly, parental technology use is closely related to that of their child. Secondly, that despite parents frequently voiced concerns about the nature and extent of their child’s mobile phone use, parents themselves often engage in a number of unsafe internet behaviors and excessive phone use in the home environment. Given parental concerns about the possible negative impact of technologies, parental awareness should be raised about the influence of their behavior in the context of internet safety along with the adoption of good digital literacy practices.

Future research should therefore draw upon psychological factors and contextual factors to develop understanding and inform future practice. It is essential to raise parental awareness of the power of their own smartphone behaviors as it is often a case of “Do as I say rather than as I do” (Terras et al., 2016) which our review indicates is detrimental to their regulation of smartphone use.

## Author Contribution

Both MT and JR authors reviewed and synthesized the research and drafted and made revisions on the paper.

## Conflict of Interest Statement

The authors declare that the research was conducted in the absence of any commercial or financial relationships that could be construed as a potential conflict of interest.
